# Brain metastasis from male breast cancer treated 12 years ago

**DOI:** 10.11604/pamj.2016.23.31.8665

**Published:** 2016-02-06

**Authors:** Tariq Namad, Rachid Ammor

**Affiliations:** 1 Medical Oncology, Military Hospital My Ismail, Meknes, Morocco; 2 Neurosurgery, Military Hospital My Ismail, Meknes, Morocco

**Keywords:** Male breast cancer, brain mass, metastatic breast cancer

## Image in medicine

Male breast cancer is an uncommon disease that has been the focus of limited researches. Its etiology is unclear, but hormonal levels may play a role in the development of this disease. Our case is a 84 year old patient treated for breast cancer 12 years ago, it was an infiltrating ductal carcinoma classified pT2 N0 M0 with hormone receptor positive (HR +), treated with surgery, adjuvant chemotherapy, radiation therapy and 5 years of endocrinotherapy (tamoxifen 20mg 1 tablet daily continuously). Clinical control of our patient was normal within 11 years and 10 months. A month ago, the patient had headache and vomiting complicated by the sudden onset of left hemiplegia. The brain MRI showed a huge right temporal process with a shift of the midline structures (figure). A biopsy was also performed and demonstrated a cerebral relapsed breast primitive with the same disease profile (HR positive and HER2 negative). Brain metastases traditionally occur in 10-16% of metastatic breast cancer patients and are associated with a bad prognosis. The development of brain metastases has been associated with young age, and tumors that are estrogen receptor negative, HER2 positive or of the basal phenotype. However, we can also have brain metastases of a primary breast cancer in elderly patients even with pathological and immunohistochemistry different profiles regardless of gender.

**Figure 1:**
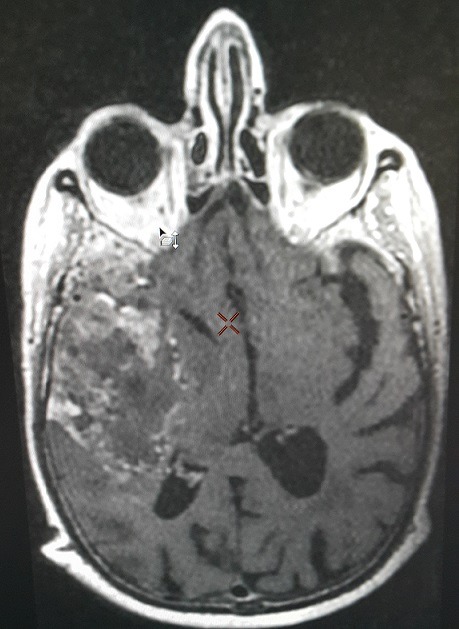
Brain MRI (axial section, injected T1-weighted sequence) showing a huge right temporal mass, taking contrast heterogeneously, with a shift of the midline

